# Mortality and Morbidity of Human Metapneumovirus Infection in the Pre-COVID-19 Era: The Value of the Charlson Comorbidity Index on Outcome Prediction

**DOI:** 10.7759/cureus.52321

**Published:** 2024-01-15

**Authors:** Merita T Shehu, Arturo Pascual, Piotr Kapinos, Marc Y El Khoury

**Affiliations:** 1 Internal Medicine, Westchester Medical Center, New York Medical College, Valhalla, USA; 2 Infectious Disease, Westchester Medical Center, New York Medical College, Valhalla, USA

**Keywords:** hospitalization, morbidity and mortality, outcome evaluation, charlson comorbidity index, human metapneumovirus

## Abstract

Introduction: Human metapneumovirus (HMPV) is an important cause of seasonal respiratory tract infections, mainly in children and immunocompromised adults. The use of the Charlson Comorbidity Index (CCI) to predict outcomes in hospitalized patients has been validated in several settings.

Objective: This study aims to describe the clinical characteristics of adult patients with HMPV infection and evaluate the value of the CCI in predicting outcomes in patients with acute HMPV infections requiring hospitalization.

Method: This is a single-center case-series study of hospitalized patients with HMPV infection in 2017.

Results: Twenty-two adult patients with a mean age of 65 years were reviewed. The mean CCI was 4.6±2.6. The overall mortality was 22%. An abnormal chest X-ray (CXR) was reported in 15 patients. CCI was not different between survivors and non-survivors. Non-survivors were more likely to have abnormal CXR and a higher fever at the time of diagnosis, required mechanical ventilation, or had other concomitant infections.

Conclusion: The average CCI was 4.5, which was not significantly different between survivors and non-survivors. The mortality rate was elevated by 22% and was likely associated with admission to the ICU as well as the presence of another concomitant infection.

## Introduction

Human metapneumovirus (HMPV) is a paramyxovirus that causes respiratory tract infections in humans. It was discovered in 2001 in the Netherlands. Infection is most common in the winter and spring in temperate climates. This pathogen commonly affects children; however, adults are affected as well. HMPV is like respiratory syncytial virus (RSV) and has been frequently compared to it. Transmission from person to person is thought to be through nasopharyngeal secretions, as per Peiris et al., who found HMPV virus RNA in the nasopharyngeal secretions of 32 children admitted with acute respiratory symptoms [1]. The incubation period is thought to be five to six days in most cases of an HMPV outbreak in two long-term care facilities, as per a CDC report [2]. Clinical manifestations vary in severity; in the adult population, they commonly include cough, nasal congestion, rhinorrhea, dyspnea, hoarseness, wheezing, and fever, to a lesser extent. Laboratory studies are generally nonspecific but may include leukocytosis, a rise in transaminases, or worsening kidney function. Chest radiographs are most often normal. The clinical course of HMPV infection is generally mild and may be asymptomatic. Young children and geriatric patients with comorbidities generally have the most severe clinical manifestations, as Boivin et al. found when studying 37 patients, of whom 12 were children [3]. Children have traditionally been studied more extensively since diseases are more common in this population. The increased number and severity of underlying comorbidities may predispose to a worse outcome in adults. HMPV has a higher prevalence among HIV-infected individuals, as Groome et al. reported after studying 593 children and 119 adults [4]. Hoellein et al. found that it leads to devastating illnesses in patients with hematologic malignancies. He studied 118 patients who received hematopoietic cell transplants and noticed that the use of corticosteroids or low lymphocyte counts were risk factors for the progression of disease [5]. HMPV has been demonstrated to affect type 2 alveolar epithelial cells and bronchial cells in macaques. It has been shown to cause alveolar and interstitial inflammation, as per Schildgen's medical review of HMPV and its pathogenesis in the Clinical Microbiology Journal [6]. Because HMPV tends to occur in the winter and early spring, it may frequently be associated with a coinfection with another virus or bacteria. Walsh et al. studied 1,386 hospitalized patients and identified HMPV infection in 8.5% of them, and 22% of the HMPV-infected patients were found to have another viral infection [7]. HMPV is detected much more efficiently after the increasing popularity of respiratory viral multiplex PCR. Nawrocki et al., who studied the effect of five social distancing policies on non-COVID-19 infections, noticed that social distancing policies during the period of January-May 2020 reduced the incidence of non-COVID-19 infections, including HMPV [8].

In this manuscript, we will discuss the results and outcomes of all adult patients who required hospitalization for HMPV infection in 2017. We will describe the spectrum of clinical manifestations and try to determine the usefulness of the Charlson Comorbidity Index (CCI) in the prediction of mortality in acutely ill patients infected with HMPV. The CCI has been used since 1987 to predict the risk of death in patients with certain underlying chronic diseases. It was tested for its ability to predict the risk of death from comorbid diseases in a 10-year period; the higher the score, the higher the cumulative mortality. We hypothesized that infection due to HMPV may be associated with greater morbidity and mortality, especially in patients who have a high CCI. Our hospital serves the lower Hudson Valley of New York State.

This article was previously posted in Authorea in June 2023.

## Materials and methods

This is a single-center retrospective chart review study of all adult patients aged 18 years or older who were diagnosed with HMPV on admission or during their hospital stay during the winter and spring of 2017. The study protocol was approved by the Institutional Review Board of New York Medical College (approval number). Inclusion criteria included a positive molecular test for HMPV, an age greater than 18 years old, and a clinical diagnosis of upper respiratory illness (URI) or lower respiratory illness (LRI). URI was defined as having one of the following respiratory symptoms: cough, sore throat, rhinorrhea, or nasal congestion. LRI was defined as having cough, dyspnea, or chest pains; an increase in oxygen requirements with or without any of the URI symptoms; evidence of wheezing, rhonchi, crackles, egophony, or decreased air entry on pulmonary auscultation; or the presence of pulmonary infiltrates on a chest radiograph. Patients were excluded if the positive PCR was not associated with any documented respiratory illness. Patients were counted once, even if a repeat molecular test was positive during a subsequent readmission within the same season.

A complete list of all the patients who had a positive nasal respiratory viral PCR for HMPV was obtained from the microbiology laboratory. Each patient was given a study number, and the data was deidentified after collection with an identification list secured safely in the research office. Medical records were reviewed, and patients who met the inclusion criteria were included. Demographics, clinical, laboratory, radiographic, treatment, and outcome data were collected at the time of admission or the time when the multiplex PCR was taken for patients who developed the infection during hospitalization. Clinical data that was collected included symptoms at the time of presentation such as cough, dyspnea, sore throat, fever, myalgia, use of alcohol, use of corticosteroids or other immunosuppressive medications, smoking, comorbidities, sick contacts, duration of illness prior to diagnosis, vital signs as well as physical lung exam, presence of concurrent or subsequent superinfection, and antibiotics used and duration. Laboratory data included CBC and comprehensive metabolic panel arterial blood gas within 24 hours of diagnosis. Initial and follow-up chest radiograph results were collected too. The CCI score was calculated for every patient and recorded. Total and intensive care unit length of stays and whether the patients survived or died were also collected.

The initial data was collected on an Excel sheet (Microsoft Corporation, Washington, USA) and transferred to SPSS Statistics version 27 (IBM Corp. Released 2020. IBM SPSS Statistics for Windows, Version 27.0. Armonk, NY: IBM Corp.) for statistical analysis. The collected data were summarized using descriptive statistics, mean, median, and standard deviation for continuous variables and numbers and percentages for categorical variables. To assess if there is a significant relationship between two categorical variables among survivors and non-survivors, we used the Pearson Chi-Square. Given the small number of patients included in each group, Fisher's exact test analysis was also done. Differences in means among continuous variables were analyzed using the Student's t-test. A p-value of less than 0.05 was considered significant.

## Results

In this study, we found 22 hospitalized adult patients who were diagnosed with HMPV infections, 20 of them community-acquired and two nosocomial. The clinical characteristics of those patients can be reviewed in detail in Table 1. The mean age was 65, and 50% were female. The mean duration of illness prior to diagnosis was 4.5 days. Most patients complained of cough and dyspnea, 85.7% and 71%, respectively. Fever and rhinorrhea were reported in only 36.3% and 38% of the cases, respectively. Sore throat and myalgia were even less frequently reported in 14.2% and 23.8%, respectively. The mean temperature at the time of diagnosis was 98±1.5. Sixty-eight percent of the patients had an abnormal chest X-ray (CXR). Leucocyte count was usually normal, with a mean of 8.4±4.1 x103 cells/µL. The mean CCI was 4.6±2.6 (1-11). The overall length of hospital stay ranged from two to 28 days, with an average of 10 days (excluding the two nosocomial cases). About 36.3% (8/22) of patients required care in the ICU for an average length of stay of 3±5.8 days (excluding one nosocomial case diagnosed in the ICU). Twenty-two percent (5/22) of patients died (Table 1). Four of them were female. Among the 15/22 patients who had abnormal CXR, most 13/15 had bilateral infiltrates (five were alveolar versus six interstitial and two mixed). All patients who died had an abnormal CXR with a trend of statistical significance. Compared to the group of patients who survived, the group of patients who died did not have a significant statistical difference in terms of CCI, age, ethnicity, smoking, or alcohol use. Patients who died were more likely to have a higher fever at the time of diagnosis, but other vital signs such as heart rate, blood pressure, oxygenation, and respiratory rate were not significantly different among the two groups. Patients who were admitted to the ICU either required mechanical ventilation or were treated for other concomitant infections. They were more likely to die and had longer ICU lengths of stay (Table 2). Patients who died were all infected in the spring, and 4/5 were on mechanical ventilation. They had severe concomitant diseases such as malignancy (2), liver failure and candidemia (1), heart failure with pulmonary hypertension (1), and extensive burns with bacteremia (1). Apparently, they had more severe respiratory failure and weaker immune systems.

**Table 1:** Clinical characteristics of the study patients SD: standard deviation, n: number of patients, N: total number of patients, F: degree Fahrenheit, O2: oxygen, CCI: Charlson comorbidity index, ICU: intensive care unit * excluding two nosocomial cases, ** excluding one case diagnosed in the ICU

Characteristics	Mean±SD (range) or n/N (%)
Age, years	65±17 (33-95)
Sex(n), males	11/22 (50%)
Duration of illness prior to diagnosis, days	4.5±3.5 (1-4)
Reported fever	8/22 (36.3)
Rhinorrhea	8/21 (38%)
Cough	18/21 (85.7%)
Dyspnea	15/21 (71.4%)
Sore throat	3/21 (14.2%)
Myalgias	5/21 (23.8%)
CCI	4.6±2.6 (1-11)
Abnormal chest X-ray	15/22 (68.2%)
Temperature, F	98±1.5 (96.9-101.8)
O2 saturation, %	95±2 (90-99)
Leukocytes x 10^3^/µL	8.4±4.1 (2.6-17)
Duration of steroids, days	7±7.7 (0-24)
Requiring ICU care	8/22 (36.3)
Hospital days	10±8.7 (2-28)*
ICU days	3±5.8 (0-23)**
Died	5/22 (22%)

**Table 2:** Clinical, laboratory, and radiographic characteristics of patients with HMPV at presentation stratified by outcome SD: standard deviation, N: total number of patients in a category, n: number of patients, %: percentage, CCI: Charlson comorbidity index, SBP: systolic blood pressure, HR: heart rate, RR: respiratory rate, F: degree Fahrenheit, EtOH: ethyl alcohol: ICU: intensive care unit, pO2: partial pressure of oxygen, NS: nonsignificant, HMPV: human metapneumovirus * excluding two nosocomial cases, ** excluding one case diagnosed in the ICU

Characteristics	Alive N=17 mean±SD or n (%)	Died N=5 mean ±SD or n (%)	Student's T-test p-value	Pearson chi-square p-value/Fisher’s exact test p-value
Age, years	65±18	65±15	NS	-
Sex, male	10 (58%)	1 (20%)	-	NS
Race, non-White	5 (29%)	1 (20%)	-	NS
Smoking	3 (17.5%)	3(60%)	-	NS
EtOH use	4 (23.5%)	1(20%)	-	NS
CCI	4.5±3	5±3	NS	-
CCI>4	6 (35%)	2 (40%)	-	NS
SBP, mmHg	121±19	120±24	NS	-
HR, beats/min	89±17	98±19	NS	-
RR breathes/min	21±3	23±7	NS	-
Temperature, F	98±1.2	99.7±1.9	0.025	-
O2 saturation%	96±2	96±3	NS	-
Hospital stays, days*	8.3±7.5	16.5±10.7	NS	-
Requiring ICU care	4 (23.5%)	4 (80%)	-	0.021/0.04
ICU stay, days**	1.6±3.2	9.5±10	0.011	-
Duration of illness prior to diagnosis, days	4±3	6±4	NS	-
pO2, mmHg	67±15	60±15	NS	-
Leukocytes x10^3^/µL	8.2±3.8	9.1±5.7	NS	-
Mechanical ventilation	4 (23.5%)	4 (80%)	-	0.021/0.04
Acute kidney injury	3 (17.5%)	1 (20%)	-	NS
Dyspnea on presentation	11 (64.7%)	4 (80%)	-	NS
Presence of infiltrates on chest radiography	10 (58%)	5 (100%)	-	0.082/0.13
Corticosteroid use	5 (29%)	3 (60%)	-	NS
Concurrent infections	2 (11.7%)	3 (60%)	-	0.024/0.05

## Discussion

Our study is the first to evaluate the predictive value of CCI in adult hospitalized patients with acute respiratory infections secondary to HMPV. CCI was initially proposed in 1987 to predict one-year mortality in hospitalized patients. It was created by Charlson et al., who also tested its ability to predict the risk of death from comorbid disease in a 10-year follow-up cohort [9]. CCI has been validated in many studies. Ather and Nazim validated it in patients with partial or complete nephrectomy for renal cell carcinoma after a 39±5-month follow-up period. His study of 157 cases of nefrectomy showed that not only the size and grade of the tumor predicted survival, but also the CCI had predictive value [10]. Froehner et al. validated it in patients with post-radical cystectomy who underwent radical cystectomy for muscle-invasive or high-risk non-muscle-invasive urothelial or undifferentiated bladder cancer after a five-year follow-up period. He studied 1,337 patients and compared CCI with a modified self-administrable comorbidity index that was based on standard sets for neoplastic diseases [11]. CCI was also found to predict outcomes in internal medicine-related complications post-hip arthroplasty after a mean 27-month follow-up period by Schmolders et al. [12]. He studied 142 patients post-hip arthroplasty and noticed that CCI influenced surgical and internal complications and the need for intensive care and hospital stays. CCI proved to be good at predicting long-term functional outcomes for patients with stroke in the study done by Tessier et al. [13]. He compared the predictive value of CCI in functionality outcome with two other indexes in more than 600 post-stroke events and concluded that CCI was valid to predict functional outcome. Frenkel et al. also proved that CCI independently predicts short- and long-term mortality in acutely ill hospitalized elderly adults in a prospective cohort study. He studied 1,313 elderly patients 65 and older who were acutely ill and required hospitalization, following them for five years [14]. An elevated CCI was predictive of high mortality in 59 hospitalized patients with COVID-19 and concomitant end-stage renal disease on hemodialysis in a retrospective study by Valeri et al. [15].

A systematic review and meta-analysis of hospitalized patients with COVID-19 by Kuswardhani et al. showed that a CCI higher than 3 was associated with increased mortality, severe diseases, and poor outcomes [16]. The risk of mortality increased by 16% for each increase in CCI. Kuswardhani et al. recommended using CCI for risk stratification in hospitalized patients with COVID-19 [16]. There was no significant association between CCI and an increased risk of in-hospital mortality in our case series study; however, both the survivors and dead patients had an elevated CCI. In the study by Setter et al., the comorbidity scores used to assess mortality risk in hospitalized patients with severe acute respiratory infections displayed poor results. The age-adjusted CCI score predicted in-hospital mortality. HIV infection was associated with mortality and considered a marker of the severity of the disease [17]. In that study, most patients studied had influenza pneumonia, and a few had HMPV. The low number of patients in our study precludes us from drawing strong conclusions regarding the value of CCI in the prediction of in-hospital mortality. Of note, most of the patients had a high CCI with a mean of 4.6, which explains the high mortality rate of 22%, which is not unusual for this high CCI score. A 50% mortality rate was reported by Boivin et al. in an outbreak among elderly residents of a long-term care facility (LTCF) infected with HMPV [18]. This high rate even exceeds that related to COVID-19 in LTCF, which was reported recently to be 14% according to the Centers for Medicare and Medicaid Services data [19]. It is worth noting that unlike COVID-19, HMPV, as Williams et al. found in their study, causes severe disease in young children ranging from croup, asthma exacerbations, bronchiolitis, and pneumonia, with a peak age ranging from five to 22 months old, and is relatively older than those with RSV [20]. Unlike infections due to SARS-CoV2, HMPV infections are seasonal, with winter epidemics occurring from December to April in the northern hemisphere at the same time or just after RSV epidemics. This is likely due to the lower transmissibility of HMPV, which is usually transmitted by contact with contaminated surfaces, as opposed to SARS-Cov2, which is more transmissible, mainly via respiratory droplets and contact, as reported by Meyerowitz et al., and possibly dominantly airborne, as Greenhalgh et al. concluded after their scientific analyses [21-22].

In our study, we noted that patients requiring ICU care and mechanical ventilation and those with bacterial or fungal superinfection had worse outcomes and were more likely to die, a phenomenon seen by Feldman and Anderson in patients with influenza and COVID-19 as well [23]. A high fever and abnormal CXR may indicate a worse outcome. The CXR findings in our patients were like those seen in patients infected with influenza or RSV lower respiratory tract infections. These radiographic features in patients with HMPV are different from those described by Wong et al. in patients with COVID-19, where peripheral distribution is a common finding [24]. Other features that distinguish HMPV infection from the COVID-19 Omicron variants are the presence of cough and dyspnea in most of the patients (85.7% and 71%, respectively). Rhinorrhea with nasal congestion, sore throat, and myalgia were even less frequently reported in 38%, 14.2%, and 23.8%, respectively, while Menni et al. reported that 70% of patients with Omicron variants of SARS-Cov2-related infections present with sore throat, rhinorrhea nasal congestion, and myalgia [25]. We are aware of the limitations of the study, such as the low number of patients and the fact that it is a retrospective study in one academic hospital center. Due to the small number of patients in each arm, this study has low statistical power, requiring the use of Fisher's exact test in addition to Pearson chi-square.

## Conclusions

We described the clinical characteristics and outcomes of HMPV infection in 22 adult patients in an inpatient setting and how it differs from COVID-19. Many patients had comorbid conditions with an average CCI of 4.5, which was not significantly different between survivors and non-survivors. The mortality rate was elevated at 22% and was significantly associated with admission to the ICU as well as having another concomitant infection. The majority of non-survivors were female. Given the low statistical power of our study, we cannot draw strong conclusions from our results; thus, larger studies in adults are warranted to better understand which factors are more likely to increase morbidity and mortality in this patient population.
